# Constructive and destructive interparental conflict, parenting entropy, and child ADHD symptoms

**DOI:** 10.1017/S0954579426101655

**Published:** 2026-07-13

**Authors:** Zhi Li, Melissa Sturge-Apple, Patrick Davies

**Affiliations:** 1 Mt. Hope Family Center, University of Rochester, USA; 2 Department of Psychology, University of Rochester, USA; 3 Warner School of Education, University of Rochester, USA

**Keywords:** child ADHD symptoms, family, interparental conflict, parenting entropy, parenting Unpredictability

## Abstract

This multi-method, multi-informant, three-wave longitudinal study sought to examine how maternal and paternal parenting entropy might operate as indirect factors in the link between interparental conflict and the development of child attention-deficit/hyperactivity disorder (ADHD) symptoms during early childhood. Participants were 235 families with a young child (Wave one: *M*_child age_ = 2.97 years, *N*_girls_ = 130, Child race/ethnicity: 56.2% White, 21.3% African American, 16.2% Mixed race) followed over three annual waves. We observed constructive and destructive interparental conflict during an interparental conflict discussion task and created parenting entropy based on multiple aspects of molar-rated parenting behavior during parent–child interaction. Child ADHD symptoms were reported by both parents over time. Findings indicated that only greater maternal parenting entropy operated as the indirect factor linking interparental conflict with elevated risks for child ADHD symptoms over time (Association with Maternal Parenting Entropy: Constructive conflict: *β* = −0.23, Destructive conflict: *β* = 0.17; Maternal parenting entropy in association with Child ADHD symptoms: *β*s range: [0.16, 0.17]). Follow-up tests comparing the role of constructive vs. destructive forms of interparental conflict highlighted the role of constructive interparental conflict. Findings highlighted potential targets to mitigate the risks associated with unpredictable parenting behavior.

Attention-deficit/hyperactivity disorder (ADHD) symptoms involve behavioral manifestations of inattention, as well as hyperactivity (i.e., excessive motor activity) and impulsivity (Epstein & Loren, 2013). Prior work has highlighted the role of exposure to early adversity in the elevated risk of developing ADHD symptoms, commonly adopting a cumulative risk approach that assumes the greater the risk exposure, the higher the subsequent risk (e.g., Evans et al., 2013; Humphreys et al., 2019). The present study is guided by the evolutionary dimensional approach to environmental adversity (e.g., Ellis et al., 2009, 2022) and seeks to evaluate how exposure to unpredictable childhood experiences stemming from parenting and family environments may shape children’s development of ADHD symptoms over time. In addition, we also aim to evaluate the antecedents of unpredictable maternal and paternal parenting by examining the role of different forms of interparental conflict.

## Unpredictability in parenting: parenting entropy approach

Early life adversity is linked to elevated risks for ADHD symptoms in children. Much of the prior work in this area commonly conceptualized the association through a cumulative-risk, dose–response model, assuming that greater adversity exposure is associated with higher subsequent risks of developing ADHD symptoms (e.g., Evans et al., 2013; Humphreys et al., 2019). To gain greater precision in understanding the mental-health sequelae of early-life adversity, Ellis and colleagues (2009, 2022) promoted the evolutionary dimensional model of environmental adversity, which emphasizes the qualitatively distinct characteristics of environmental adversity. One of the critical dimensions of adversity is the unpredictability of the context, which refers to random variations in resources or risks posed by the external environment that the developing person cannot reasonably foresee or control. According to the evolutionary perspective, exposure to environmental unpredictability may shift development towards a riskier end, as indicated by some ADHD symptoms, including greater impulsivity, diminished focused attention, and a lack of behavioral inhibition (e.g., Del Giudice, 2014; Del Giudice & Haltigan, 2023). To this end, various empirical studies have confirmed the association between exposure to unpredictability and elevated ADHD symptoms in children (e.g., Agnew-Blais et al., 2022; Li & Lansford, 2018).

Notably, according to Ellis and colleagues (2022), unpredictability may originate from the proximal rearing context and has crucial implications for child development. Towards this, prior research has examined proximal unpredictability, focusing on various aspects of parenting and family unpredictability. For instance, prior work has focused on unpredictability in maternal sensory input (e.g., Davis et al., 2017, 2019; Holmberg et al., 2022) or maternal mood entropy (i.e., fragmented, unpredictable patterns in maternal mood; Glynn et al., 2018; Howland et al., 2021; Jirsaraie et al., 2024). Others have examined disorganized, out-of-context parenting behaviors during parent–child interactions (e.g., Obsuth et al., 2014). Emerging research has also investigated fluctuations in parenting behavior measured across different time scales (e.g., year-to-year, moment-to-moment; Li et al., 2023; Lippold et al., 2018; Zheng & McMahon, 2022) and contexts (e.g., Platts et al., 2025). Finally, recent studies have captured caregiver unpredictability specifically during dyadic parent–child interactions (e.g., Li et al., 2025; Ugarte & Hastings, 2025). Despite the critical contributions of this line of work, the quantification of parenting unpredictability commonly involves repeated and intensive assessments of parenting behavior (e.g., Davis et al., 2017, 2019; Holmberg et al., 2022; Li et al., 2023; Lippold et al., 2018; Zheng & McMahon, 2022) or parent–child interactions (Li et al., 2025; Ugarte & Hastings, 2025), which is a resource- and time-intensive process.

To address this issue, Glynn and associates (2018) proposed quantifying maternal mood patterns based on mothers’ responses to standardized mood questionnaires using Shannon’s entropy, enabling the assessment of unpredictable patterns of maternal mood with a single measurement occasion rather than repeated assessments. In particular, an entropy score was calculated for each participant by applying Shannon’s entropy formula to the relative frequency distribution of their responses across all questionnaire items, after appropriate reverse scoring. As such, the mood entropy measure reflects the degree to which a participant responds inconsistently to questions, with higher entropy indicating more unpredictable, scattered responses across all items. Glynn and colleagues (2018) have demonstrated that the mood entropy measure exhibited excellent discriminant (i.e., mood entropy not related to entropy in physical activity) and convergent validity (i.e., mood entropy was correlated with parent mood variability measured via ecological momentary assessment). Furthermore, maternal mood entropy exhibited great predictive validity, such that greater prenatal maternal mood entropy was associated with poorer child cognitive development (Howland et al., 2021), greater psychopathology (Davis & Glynn, 2024; Glynn et al., 2018), and weakened and inflexible neural circuit development (i.e., salience network, Jirsaraie et al., 2024).

Building on the promise of the mood entropy approach, this study applies it to parent–child interactions, capturing the unpredictability and disorganization of parenting behavior. More specifically, we assessed the entropy of molar ratings of several aspects of parenting behavior during parent–child interactions, all of which were presumed to reflect a common underlying dimension of parenting practices (e.g., general unsupportiveness). A parent with low parenting entropy, and thus low unpredictability and disorganization, should behave consistently across various parenting behavior indicators. In contrast, parents who are highly unpredictable and disorganized may receive ratings that are highly scattered and diverse across various parenting behavior indicators. Thus, the entropy in parenting behavior is designed to capture the overall patterns of parenting behavior, as well as its salience and level.

## Constructive and destructive interparental conflict and parenting entropy

Whereas existing work has largely focused on the developmental consequences of exposure to unpredictability, little research has examined its antecedents. Identifying these precursors may provide valuable guidance for treatment and intervention programs aiming to disrupt the risk cascade of unpredictability that eventually impacts children. Guided by family system theory, we aim to examine how interparental conflict is associated with parenting entropy. Family system theory highlights the interdependence of different family subsystems (e.g., marital and parent–child subsystems), such that the quality of interaction within one subsystem may shape the others (Cox & Paley, 1997; Cox et al., 2001). According to the spillover hypotheses, the quality and dynamics of the interparental relationship may spillover into the parent–child relationships, affecting the quality of parenting (Easterbrooks & Emde, 1988; Grych, 2002). Whereas the vast majority of prior research focused on the spillover of negative dynamics between the two subsystems (e.g., spillover of hostility and destructive interparental conflict), more recent work has highlighted the distinct forms of interparental conflict and emphasized the importance of examining both constructive and destructive dimensions (e.g., Davies et al., 2012). In particular, destructive conflict encompasses the negative and hostile forms of interparental conflict, including hostility, physical and verbal anger, and withdrawal, and has been shown to elicit harsh and less warm and supportive parenting in both mothers and fathers (e.g., Krishnakumar & Buehler, 2000; Sturge-Apple et al., 2009). In contrast, constructive conflict involves more positive forms of conflict, characterized by support, problem-solving, and the expression of physical and verbal affection between partners as they resolve disagreements (e.g., Davies et al., 2012). Constructive interparental conflict has been linked to more parental warmth and support, and less problematic forms of parenting in both parents (e.g., Kopystynska et al., 2020; McCoy et al., 2013; Warmuth et al., 2020).

Despite this, to our knowledge, no research has investigated how interparental conflict may shape parenting entropy. Findings on inconsistent discipline may provide some guidance. This is because both parenting entropy and inconsistent parenting reflect parental disorganization and unpredictability from the children’s perspective, and potentially a failure to maintain consistent parenting strategies and standards across different moments and contexts. In this regard, prior research has documented the association between both constructive and destructive interparental conflict and inconsistent parental discipline (e.g., McCoy et al., 2013; Warmuth et al., 2020). For instance, in a study that followed families with children over three annual measurement occasions (i.e., from child age six to eight), Warmuth and colleagues (2020) examined how interparental conflict, measured via survey and observation of interparental interaction (i.e., resolving a conflict) at the first time point, was linked to parental inconsistent discipline one year later. Constructive forms (e.g., cooperation, problem solving, support) and destructive forms (e.g., hostility, verbal aggression, verbal and nonverbal anger) of interparental conflict were associated with lower and higher inconsistent discipline, respectively, one year later, for both mothers and fathers. In addition, although not explicitly contrasted, the result suggested that the effect size for constructive conflict was stronger than that for destructive conflict. Taken together, while direct evidence is limited, prior research has provided theoretical and empirical grounding for investigating how both constructive and destructive interparental conflict may be associated with parenting entropy.

## Parenting entropy and child ADHD symptoms

This study also aims to investigate how parenting entropy may influence the development of children’s ADHD symptoms. Despite limited direct evidence linking parenting entropy and child ADHD symptoms, prior work has documented that exposure to more unpredictable parenting over time, measured via ecological momentary assessment, was associated with greater child ADHD symptoms (e.g., Li & Lansford, 2018). Furthermore, disorganized and unpredictable parenting may increase the risks for developing ADHD symptoms via several plausible mechanisms. From an evolutionary developmental perspective, unpredictable environments signal that the future is uncertain, which may prompt behavioral and neurobiological adaptations that prioritize immediate monitoring of the environment over long-term goal persistence. First, unpredictable parenting may disrupt children’s development of effortful control (e.g., Davis et al., 2019; Davis & Glynn, 2024; Holmberg et al., 2022), thereby undermining their ability to effectively regulate impulses and attention. In turn, numerous studies have documented the role of diminished effortful control in children’s development of ADHD symptoms (e.g., Atherton et al., 2020; Martel & Nigg, 2006). For instance, in two longitudinal cohorts (i.e., one US and one Finland cohort) both followed children from infancy to childhood, Davis and colleagues (2019) quantified parenting unpredictability during a parent–child free-play interaction during infancy, and measured child effortful control via both parent-report and a behavioral task (i.e., Flanker task) at various ages during early to middle childhood in the two cohorts. Children exposed to greater maternal unpredictability during infancy showed lower effortful control, and this effect persisted at least until age five in the Finnish cohort (Davis et al., 2019; Holmberg et al., 2022) and until age 9.5 in the US cohort (Davis et al., 2019).

Second, exposure to unpredictable parenting has been associated with deficits in general cognitive development, as demonstrated by prior studies (e.g., measured via Bayley Scales of Infant Development at Age 2; Davis et al., 2017). Thus, although this study did not differentiate the role of parenting unpredictability across specific aspects of cognitive performance, several cognitive functions assessed by the Bayley Scale (e.g., the ability to pay and sustain attention; the problem-solving ability that requires holding the information to achieve goals) may have important implications for ADHD symptoms. Specifically, when environmental inputs are erratic and unpredictable, the developing cognitive system may become “tuned” toward a more reactive rather than proactive style of engagement, manifested as distractibility and impulsivity characteristics of ADHD. Third, an emerging line of work has highlighted a link between exposure to parenting unpredictability and impaired brain circuit development and maturation (e.g., Davis & Glynn, 2024; Glynn & Baram, 2019; Jirsaraie et al., 2024). This research suggests that patterns of early-life signals are critical drivers of neurodevelopment and that unpredictable inputs may disrupt the refinement of fundamental circuits. For instance, this line of research suggests that prenatal exposure to maternal mood entropy is associated with a weakened and inflexible salience network (Jirsaraie et al., 2024). In addition, exposure to unpredictability may be associated with altered brain circuits for cognition and emotion, which may manifest as deficits in self-control and executive function. Collectively, these neurodevelopmental alterations may in turn create a vulnerability for elevated ADHD symptoms. Taken together, these findings suggest that parenting entropy may be associated with increased ADHD symptoms in children.

## The present study

This multi-method, multi-informant, longitudinal study seeks to investigate how constructive and destructive forms of interparental conflict are associated with the development of maternal and paternal parenting entropy, and in turn, the development of child ADHD symptoms. To obtain a rigorous, temporally ordered test of the indirect pathways that control for the initial levels of parenting entropy and child psychopathology, we utilized a three-wave longitudinal design with repeatedly measured parenting entropy at the first and second waves, and child ADHD symptoms at the first and third waves. Parenting entropy was quantified using Glynn and colleagues’ (2018) approach, which is based on maternal mood entropy, utilizing molar ratings of multiple aspects of parenting behavior assumed to reflect the same underlying parenting dimension (i.e., general unsupportiveness) during mother–child and father–child dyadic interactions. Although direct evidence on parenting entropy is limited, guided by prior research, we hypothesize that constructive and destructive forms of interparental conflict are associated with lower vs. higher levels of parenting entropy, respectively, for both parents. In turn, we hypothesize that greater parenting entropy observed in both parents is associated with greater child ADHD symptoms over time. This study makes several contributions to the literature. First, expanding the existing research on child consequences of exposure to unpredictable parenting, this study seeks to illuminate the antecedents as well as child consequences of unpredictable parenting. Second, to build on the existing methods for quantifying parenting unpredictability, which are time- and resource-demanding, we propose and examine a novel approach to measuring parenting unpredictability grounded in prior literature (Davis & Glynn, 2024; Glynn et al., 2018). Finally, to build on existing literature that mainly focuses on unpredictability in maternal parenting, this study adds to the literature by examining the role of paternal parenting entropy as well.

## Method

### Participants

Participants are 235 young children (55.3% were girls, *N* = 130), and their parents were recruited from a mid-sized city in the Northeastern United States. Recruitment happened in child-care centers, Head Start programs, local events, and through flyers and family websites. Interested families were screened and enrolled in the study if following eligibility criteria were satisfied: (a) the target child was at least three years of age; (b) the target child and the two parental figures are living together in the same household for at least the previous year; (c) the two parental figures were of the opposite sex, and at least one of the them is the biological parent to the participating child; (d) The target child does not have cognitive or developmental disabilities, and the three family members could communicate fluently in English. Families participated in laboratory visits in three consecutive years; each was scheduled roughly one year apart. At the first wave (Data collection: April 2017 to September 2018), the mean age for participants was 2.97 years old (*SD* = 0.38, Age range: [2, 4]), 33.56 years old (*SD* = 5.30, range: [20, 48]), and 36.00 years old (*SD* = 6.44, range: [19. 62]) for child, mother, and father, respectively. The age range for children was a result of accommodation for various scheduling considerations, and we provided a one-month flexibility window for family visits (i.e., one month before and after the child turned three). At the initial wave, 56.2% of the children were identified as White, 21.3% as African American, and 16.2% as mixed race. In addition, 16.2% children were identified as Hispanic or Latino ethnicity. The median level of maternal and paternal highest education was an associate’s degree and some college (< two years), respectively. The median household income fell within the range of $55,000–74,999, and 25.5% of families reported a household income below $23,000.

218 families (92.8%) completed the second wave (Data collection: June 2018–August 2019), with the mean age for participants being 3.88 years old (*SD* = 0.51, Age range: [3, 5]), 34.54 years old (*SD* = 5.29), and 37.13 years old (*SD* = 6.35) for child, mother, and father, respectively. Finally, 205 families (87.2%) completed the third wave (Data collection: June 2019–January 2021), and participants were on average 5.04 years old (*SD* = 0.55, Age range: [4, 6]), 36.06 years old (*SD* = 5.06), and 38.46 years old (*SD* = 6.28) for child, mother, and father, respectively. The study protocol was reviewed and approved by the Institutional Review Board of the University of Rochester (Title of study: Interparental Relationship and Parenting; Case number: RSRB939).

### Procedures

At each wave, families visited the laboratory for 2.5 to 3 hours. They completed interparental and parent–child interaction tasks in a designated room equipped with an audiovisual recording system. In addition, parents completed questionnaires in quiet, separate survey rooms. Parent–child interaction tasks were counterbalanced; at each wave, a randomly selected half of the families completed the mother–child interaction task first, while the other half completed the father–child task first. We measured interparental conflict at Wave 1, parenting entropy at Waves 1 and 2, and child ADHD symptoms at Waves 1 and 3 (See a flowchart of the study design in Supplemental Material, Figure S1).

#### Interparental conflict discussion (Wave 1)

Parents completed the ten-minute interparental conflict discussion task (Sturge-Apple et al., 2009) at the first wave. In the task, the experimenter instructed parents that the research team was interested in understanding how parents resolve disagreements. Parents were given several minutes to write down their own topics and then work together to pick two topics to discuss. The experimenter instructed the parents to discuss the two topics in their usual manner and to try to stay on topic throughout the task. At the midpoint (i.e., after five minutes), the experimenter knocked on the door to signal the switch to the second topic. Although the procedure does not involve standardized laboratory stressors, the interparental conflict discussion paradigm offers greater ecological validity and has been widely used and validated for eliciting conflict between parents (Sturge-Apple et al., 2009). A post-discussion survey indicated that parents found the laboratory discussion similar to the conversations they typically have at home. On a 7-point Likert scale (1 = “A lot more negative,” 4 = “very similar,” 7 = “A lot more positive”), 27.2% of mothers and 35.7% of fathers rated the conflict discussion as “very similar” to real-life discussions. Additionally, 37.1% of mothers and 28.2% of fathers rated the laboratory discussion as “a little more positive,” while 2.2% and 4.4% mothers and fathers, respectively, indicated that the discussion was “a little more negative.” Furthermore, mothers and fathers did not significantly differ in their rated similarity of the laboratory discussion to their natural conflict at home (Mother rating: *Mean* = 5.12, *SD* = 1.10, Range: [1, 7]; Father rating: *Mean* = 4.93, *SD* = 1.20, Range: [1, 7]; *t*(217) = 1.60, *p* = .11).

#### Forbidden toy task (Waves 1 & 2)

At the first and second waves, families completed two five-minute dyadic forbidden toy tasks (i.e., mother–child and father–child tasks) in a counterbalanced manner (see general procedure). During the task (Vaughn et al., 1984), the experimenter placed an attractive toy in the room (at the first wave: an attractive ball pit play tent; at the second wave: a shelf of mini musical instruments, a tea set, and a Paw-Patrol truck) while informing the parents (but not the child) that the child cannot touch the toys during the task. Meanwhile, parents were occupied with an iPad flanker task, where they judged the direction of a center arrow in the presence of distracting arrows on both (left and right) sides. Finally, children were given two boring, empty Play-Doh boxes to play with during the tasks.

### Measures

#### Constructive and destructive interparental conflict (IPC, wave 1)

Maternal and paternal behavior were rated based on the System for Coding Interaction in Dyads (SCID; Malik & Lindahl, 2004) using nine-point molar scale (1 = *not at all characteristic,* 9 = *mainly characteristic*). Two independent coding teams (two coders per team) rated maternal and paternal behavior separately along the dimensions of (1) Negativity and conflict, assessing parents’ level of tension, frustration, irritation, and anger expressed towards their partner (Intraclass correlation [ICC] = 0.86[Mother]/0.78[Father]). (2) Verbal aggression, capturing parents’ exhibition of hostility and aggression towards their partner that involve insults, blaming, and patronizing statements (ICC = 0.62[Mother]/0.82[Father]). (3) Support (ICC = 0.68[Mother]/0.71[Father]), measuring the degree to which parents listen attentively to each other, being sensitive and attuned to their partner, and validate and attempt to understand the partner’s perspective, and (4) problem-solving communication (ICC = 0.64[Mother]/0.78[Father]), assessing the degree to which parents were able to discuss their own and partner’s feelings and perspectives constructively while maintaining respect, in addition to facilitating problem-solving by identifying the issues and generating non-judgmental solutions to resolve the issue. Following prior literature, we created two separate indicators for IPC: (1) Constructive IPC, involving maternal and paternal support, and problem-solving communication (Pearson et al., 2023; Swerbenski et al., 2023; Averaging the four codes, bivariate correlations were all significant, *r*s range: [.48, .87]), and (2) Destructive IPC, capturing maternal and paternal negativity and conflict, and verbal aggression (Pearson et al., 2023; Platts et al., 2024; Averaging the four codes, bivariate correlations were all significant, *r*s range: [.17, .71]). As expected, the constructive vs. destructive IPC were negatively and moderately correlated (*r* = −.57, *p* < .01), but the two dimensions of IPC remain independent (shared variance = 32.5%).

#### Parenting entropy (Waves 1 & 2)

To quantify parenting entropy, we followed Glynn et al. (2018) approach in measuring maternal mood entropy by applying Shannon’s entropy function to various molar ratings of maternal and paternal parenting behaviors separately across multiple aspects, all reflecting the same underlying dimensions of parenting (after appropriate reverse scoring). More specifically, we focused on the following parenting behavior during the forbidden toy task, each rated on a 9-point molar scale (1 = *Not at all characteristic,* 9 = *Highly characteristic*) according to the Caregiving around Discipline System (Jones-Gordils et al., 2021). Two coding teams independently coded the maternal and paternal parenting behavior, reaching reliability of each parenting behavior at each wave. (1) *Neglect & Distancing*, capturing the degree to which parents are uncaring, apathetic, unresponsive, and ignoring the child, and/or being self-focused during the task. (2) *Harsh discipline* reflects critical, harsh, disapproving, and demanding behavior toward the child’s actions or state, expressed in an irritable, impatient, or even hostile and humiliating manner. (3) *Authoritarian discipline* reflects parents’ firm, no-nonsense commands that must be followed immediately without question. In addition, several observational codes reflecting positive aspects of parenting behavior were also included (described below), but *reverse-scored before creating the parenting entropy indicators*, such that all the indicators for parenting entropy reflected the negative aspects of parenting. (4) *Technical scaffolding* refers to parents using the task as a “teachable moment” for the child, raising thought-provoking statements and questions, correcting the child’s behavior, and providing clear explanations at a level appropriate for the child’s comprehension; (5) *Sensitivity* reflecting parents sensitive and accurate perception and interpretation of child communication signals, in addition to parents’ prompt and appropriate responses to child signals (e.g., responding to child’s frustration with empathy and understanding); (6) *Reasoning/Reminding* capturing parents’ reminding children of the task rules with high-levels of reasoning (e.g., explaining that the experimenter established the rules, and child could play with the toy later if they are patient); (7) *Distracting* refers to assessing parents’ attempts to distract the child from the attractive toy and the quality of such distractions (e.g., high-quality distractions should be cognitively engaging and may often involve sensory input that can engage the child for a substantial amount of time). Importantly, given the age group of children, parental distraction is considered an age-appropriate strategy for parents to guide their child through the forbidden-toy task (Gilliom et al., 2002; Schoppmann et al., 2019; See Intraclass correlation coefficient [ICC] for reliability of maternal and paternal parenting behavior at each wave in the Supplemental Material, Table S1).

Similar to Glynn et al. (2018), after appropriate reverse scoring of several positive aspects of parenting behavior (i.e., technical scaffolding, sensitivity, reasoning & reminding, and distracting) such that higher scores all indicate more problematic parenting (e.g., greater harsh discipline, lower sensitivity), we calculated parenting entropy separately for mothers and fathers at each wave according to the following formula (Equation 1). One additional note is that at each wave and for both parents, the seven parenting dimensions after appropriate reverse scoring achieved excellent internal consistency (Maternal parenting: *α* = 0.77[Wave 1]/0.74[Wave 2]; Paternal Parenting: *α* = 0.76[Wave 1]/0.69[Wave 2]). Thus, all observational ratings of parenting behavior reflect a common underlying dimension of parenting (i.e., the overall negative parenting).

Turning to Shannon’s entropy formula, as shown in Equation 1, E represents all the possible responses of the molar ratings (i.e., 1–9 ratings for parenting behavior), and *p_e_* indicates the proportion of each response (i.e., 1–9 rating) across all parenting dimensions (e.g., Category “*3 = minimally characteristic”* being endorsed twice across the seven scales, thus, *p*_*e* = 3_ = 2/7 = 0.29. Notably, probabilities among all categories summed up to 1).
(1)

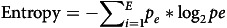




As such, Shannon’s entropy calculation quantified the degree to which parents behaved consistently across various parenting dimensions via the aggregated probabilities across each rating category (i.e., 1 to 9), with higher entropy values indicating greater unpredictability, or disorganization of parenting behavior. As an illustrative example, parents who are rated as highly supportive and nurturing across all parenting dimensions, or as highly insensitive and problematic across all dimensions, will both receive lower entropy scores, as their behavior is not scattered or unpredictable across the various parenting dimensions. Similar to Glynn et al. (2018), we normalized the entropy scores by expressing them as the percentage score of the maximum possible range.

Examination of convergent validity was performed at the first wave due to the availability of dynamically coded data (Li et al., 2025), and we examined the convergent validity in two ways: (1) testing the correlation between parenting entropy and parenting unpredictability derived via observationally coded parent–child interaction during the forbidden-toy task using 30-second epochs; (2) examining the correlation between parenting entropy and the molar rating of various aspects of parenting behavior. In (1), we calculated two indices: (1.a) parenting unpredictability, calculated as the residualized variability in parenting sensitivity over the entire course of the interaction, after accounting for systematic trends in parenting (e.g., Li et al., 2023; Zheng & McMahon, 2022); and (1.b) parent–child dyadic unpredictability, calculated as the visit entropy in the parent–child dyadic state (i.e., parent sensitivity and child dysregulation of their behavior and emotion) over the ten 30-second epochs via the state-space grid modeling approach (e.g., Li et al., 2025; Ugarte & Hastings, 2025).

We found stronger evidence for the convergent validity of *maternal* parenting entropy. First, maternal parenting entropy was marginally but positively correlated with dyadic parent–child unpredictability (*r* = .13, *p* = .05), even though it was not related to maternal unpredictability measured via the residualized variability in maternal sensitivity (*r* = .06, *p* = .35). Second, greater maternal parenting entropy was associated with lower positive aspects of maternal parenting (i.e., technical scaffolding, sensitivity, reasoning/reminding, distracting, *r*s range: [−.18, −.45], *p*s range: [<.0001, .006]), and higher negative aspects of maternal parenting (i.e., neglecting/distancing, and authoritarian parenting), *r*s range: [.18, .30], *p*s range: [<.0001, .006]; See more detailed information about correlation among parenting entropy and each parenting behavior at each wave in Supplemental Material, Table S2). Turning to paternal parenting entropy, evidence was somewhat weaker. First, paternal parenting entropy was not correlated with either dyadic father–child unpredictability (*r* = .01, *p* = .83) or paternal unpredictability measured via the residualized variability in paternal sensitivity (*r* = −.004, *p* = .95). Second, we found that greater paternal parenting entropy was negatively correlated with fewer aspects of positive parenting behavior (i.e., technical scaffolding, and marginally for distracting, *r*s range: [−.12, −.18], *p*s range: [.005, .07]). Furthermore, we found only one significant association between greater paternal parenting entropy and negative parenting behavior (i.e., authoritarian parenting; *r* = .24, *p* = .0002).

#### Child ADHD symptoms (Waves 1 & 3)

Child ADHD symptoms were measured by mother- and father-report at the first and third waves via the five-item Hyperactivity/Inattention subscale of the Strengths and Difficulty Questionnaire (Goodman, 1997). Responses were on a 3-point Likert scale (0 = “*Not True,*” 1 = “*Somewhat True,*” and 2= “*Certainty True*”; e.g., “Restless, overactive, cannot stay still for long,” “Easily distracted, concentration wanders,” “Can stop and think things out before acting”). The measure yielded good reliability at both waves (Wave 1: *α* = 0.73[mother]/0.73[father]; Wave 3: *α* = 0.82[mother]/0.75[father]), and moderate correlations across informants (Wave 1: *r* [mother- & father-report] = .38, *p* < .01; Wave 3: *r* [mother- & father-report] = .54, *p* < .01). As such, we averaged the mother- and father-report at each wave to indicate the overall child ADHD symptoms.

#### Covariate (Wave 2)

We created average parenting scores for mothers and fathers at the second wave across the seven parenting dimensions used to create the parenting-entropy indicators (i.e., reverse-scored technical scaffolding, sensitivity, reasoning/reminding, distracting; and non-reverse-scored neglect/distancing, harsh discipline, and authoritarian parenting), with higher average scores reflecting more problematic parenting behaviors. Controlling for average (negative) parenting ensures that any detected effects of parenting entropy are above and beyond the level of problematic parenting.

### Data analysis plan

To assess how parenting entropy might serve as an intermediate mechanism in the relationship between constructive and destructive IPC and child psychopathology, we ran longitudinal autoregressive models. These models included repeated measures of parenting entropy at Waves 1 and 2, child ADHD symptoms at Waves 1 and 3, and the covariate, average parenting behavior at Wave 2. The advantage of this approach is that it controls the potential confounding effects of baseline parenting entropy and ADHD symptoms at Wave 1, ensuring that any documented effects are more robust due to the stronger temporal design. More specifically, parenting entropy at Wave 2 was regressed on Wave 1 IPC, parenting entropy at Wave 1, child ADHD symptoms at Wave 1, and average parenting behavior at Wave 2. In turn, child ADHD symptoms at Wave 3 were regressed on Wave 2 parenting entropy, along with the four exogenous predictors mentioned in the previous step (e.g., Wave 1 IPC). All concurrent covariances were estimated in the model as well (e.g., Wave 1 IPC and Wave 1 child ADHD symptoms). As such, all models were saturated with all possible pathways estimated, avoiding too strong a-priori model specifications that may generate biases.

To gain greater specificity, we first ran four separate longitudinal autoregressive models: (a) Constructive vs. Destructive IPC in association with (b) Maternal vs. Paternal Parenting (Table 2 and Table 3) models. Next, we ran two additional models, one for each parent, to contrast the role of Constructive vs. Destructive IPC (Table 4). Furthermore, we follow up on significant results with additional sensitivity tests that control additional covariates (e.g., child age, gender, family income-to-needs ratio), aiming to examine the robustness of our findings. All data analyses were performed in R 4.3.2 (R Core Team, 2021). Pathway models were estimated using the lavaan_0.6-19 (Rosseel, 2012). Little’s Missing Completely at Random Test (including primary and covariates in the sensitivity analyses [e.g., child age, gender, family income-to-needs ratio] was not significant (*X* ^2^[133] = 142.53, *p* = .27). In turn, missing data were handled using full information maximum likelihood, and parameters were estimated using maximum likelihood estimation with robust standard error. Descriptive information was obtained via psych_2.4.12 (Revelle, 2024). We tested indirect effects by RMediation (Tofighi & MacKinnon, 2011) via Monte Carlo simulation.

## Results

Descriptive information and bivariate correlations for the primary study variables are presented in Table 1. Constructive and destructive IPC were moderately and negatively correlated. Maternal parenting entropy at Wave 2 was linked to lower constructive and higher destructive IPC at Wave 1. For both parents, greater averaged negative parenting at Wave 2 was linked to higher parenting entropy at Wave 2, supporting the validity of the parenting entropy constructs. Furthermore, child ADHD symptoms at both Waves 1 and 3 were at least marginally significantly linked to higher maternal parenting entropy at Waves 1 and 2. In contrast, for fathers, there was only one significant association among parenting entropy and child psychopathology: greater paternal parenting entropy at Wave 1 was linked to child ADHD symptoms at Wave 3. Maternal and paternal parenting entropy both exhibited low, but at least marginally significant, stability over the two waves. In addition, child ADHD symptoms exhibited high stability over the waves.

**Table 1.** Descriptive information and bivariate correlation among primary study variables Table 1 long description.

	1	2	3	4	5	6	7	8	9	10
1. Constructive IPC wave 1	–	–	–	–	–	–	–	–	–	–
2. Destructive IPC wave 1	−0.57**	–	–	–	–	–	–	–	–	–
3. Maternal parenting entropy wave 1	−0.09	−0.04	–	–	–	–	–	–	–	–
4. Maternal parenting entropy wave 2	−0.30**	0.20**	0.17*	–	–	–	–	–	–	–
5. Paternal parenting entropy wave 1	0.08	−0.001	−0.01	0.12^†^	–	–	–	–	–	–
6. Paternal parenting entropy wave 2	−0.05	−0.002	0.02	−0.05	0.13^†^	–	–	–	–	–
7. Averaged maternal negative parenting wave 2	−0.43**	0.17*	0.12^†^	0.22**	−0.15*	−0.02	–	–	–	–
8. Averaged paternal negative parenting wave 2	−0.28**	0.12^†^	0.05	0.18**	0.05	0.20**	0.28**	–	–	–
9. Child ADHD symptoms wave 1	−0.10	0.04	0.12^†^	0.17*	0.03	0.01	0.09	−0.06	–	–
10. Child ADHD symptoms wave 3	−0.03	0.05	0.15*	0.28**	0.14*	0.06	−0.02	0.05	0.68**	–
*N*	230	230	228	211	229	202	211	202	235	205
*Mean (SD)*	5.12 (1.89)	1.84 (1.33)	63.88 (11.26)	63.63 (11.16)	66.59 (10.53)	61.48 (10.63)	4.06 (0.91)	4.16 (0.88)	4.35 (1.96)	4.19 (2.35)
*Min*	1.25	1.00	27.23	27.23	27.23	27.23	2.43	2.00	0.00	0.00
*Max*	8.75	8.25	88.56	88.56	88.56	88.56	7.14	7.00	9.50	10.00
*Skewness*	0.01	2.24	−0.67	−0.45	−0.42	−0.49	1.44	0.86	0.32	0.24
*Kurtosis*	−1.01	5.18	0.85	0.27	0.14	0.53	1.99	0.79	−0.38	−0.59

*Note*. **p < .01, *p < .05, ^†^ *p* < .10. Highest possible entropy score for seven parenting dimensions out of 9-point molar rating: Normalized Max Entropy = [−(1/7)*log_2_(1/7) * 7]/log_2_(9) * 100 = 2.8074/3.17 = 88.56; Lowest possible range of entropy was zero (all seven dimensions of parenting were rated at the same score, but this was not present in our sample.

### Testing the indirect effects of parenting entropy

#### Mother models

**Figure 1.**
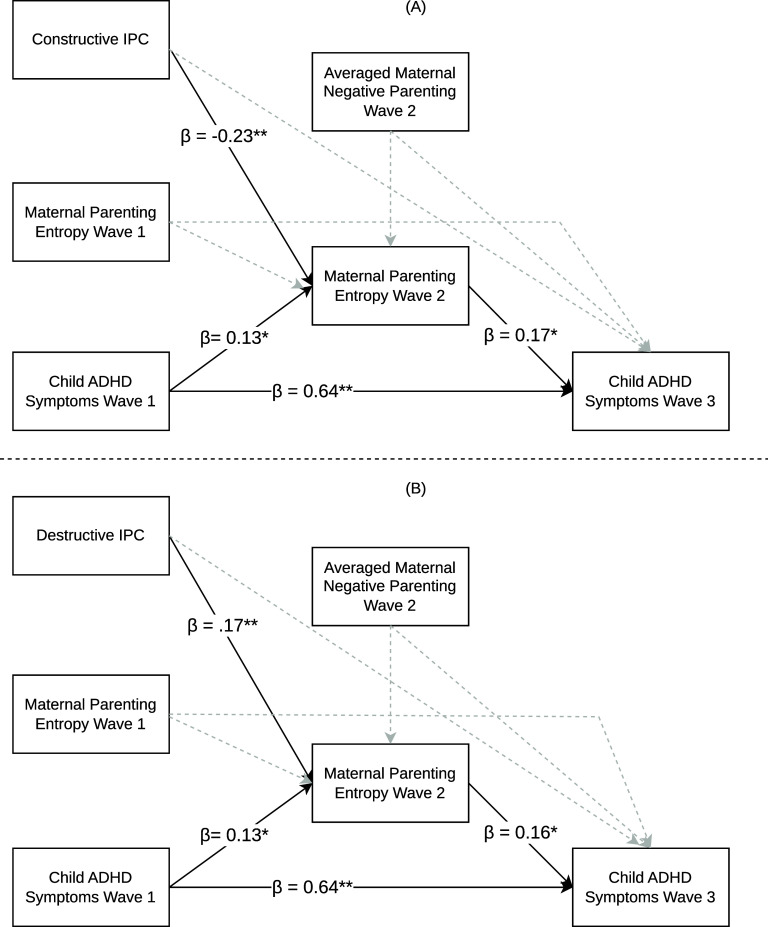
Pathway findings for IPC, maternal parenting entropy, and child ADHD symptoms. *Note.* (a): Constructive IPC Model; (b): Destructive IPC Model. *p < .05, **p < .01. Parameters presented in this figure are all standardized coefficients. While both models were specified as fully saturated (with all possible paths and covariances estimated), significant paths are plotted in solid lines, and the nonsignificant paths are plotted in light gray dotted lines.

In the constructive IPC model (Table 2, Figure 1a; See covariances of exogenous predictors in Supplemental Material, Table S6), greater constructive IPC at Wave 1 was linked to lower maternal parenting entropy at Wave 2, after controlling for entropy at Wave 1. Child ADHD symptoms at Wave 1 were linked to greater maternal parenting entropy at Wave 2. Child ADHD symptoms exhibited moderate stability across the two waves. Greater maternal parenting entropy at Wave 2 was linked to higher child ADHD symptoms at Wave 3, after controlling for child ADHD symptoms at Wave 1. The test for the indirect effect of greater constructive IPC leading to lower maternal parenting entropy, and thereby lower child ADHD symptoms over time, was significant (Estimate = −0.05, 95% Confidence Interval [CI]: [−0.10, −0.01]).

**Table 2.** Pathway findings for IPC, maternal parenting entropy, and child ADHD symptoms (*N* = 235) Table 2 long description.

	*B (SE)*	*Z*	*p*	*β*
***Constructive IPC model***				
**Child ADHD symptoms wave 3**				
Child ADHD symptoms wave 1	0.76 (0.06)	12.09	<.01	0.64
Maternal parenting entropy wave 2	0.04 (0.01)	2.43	.02	0.17
Constructive IPC wave 1	0.05 (0.07)	0.67	.50	0.04
Averaged maternal negative parenting wave 2	−0.21 (0.15)	−1.39	.17	−0.08
Maternal parenting entropy wave 1	0.01 (0.01)	1.45	.15	0.06
**Maternal parenting entropy wave 2**				
Averaged maternal negative parenting wave 2	1.23 (1.26)	0.98	.33	0.10
Maternal parenting entropy wave 1	0.11 (0.07)	1.58	.11	0.11
Constructive IPC wave 1	−1.35 (0.39)	−3.47	.001	−0.23
Child ADHD symptoms wave 1	0.73 (0.37)	1.97	.05	0.13
***Destructive IPC model***				
**Child ADHD symptoms wave 3**				
Child ADHD symptoms wave 1	0.76 (0.06)	12.04	<.01	0.64
Maternal parenting entropy wave 2	0.03 (0.02)	2.18	.03	0.16
Destructive IPC wave 1	0.04 (0.08)	0.43	.67	0.02
Averaged maternal negative parenting wave 2	−0.25 (0.14)	−1.72	.09	−0.10
Maternal parenting entropy wave 1	0.01 (0.01)	1.40	.16	0.06
**Maternal parenting entropy wave 2**				
Averaged maternal negative parenting wave 2	2.04 (1.18)	1.73	.08	0.17
Maternal parenting entropy wave 1	0.14 (0.07)	1.91	.06	0.14
Destructive IPC wave 1	1.43 (0.45)	3.20	.001	0.17
Child ADHD symptoms wave 1	0.75 (0.37)	2.05	.04	0.13

Turning to the destructive IPC models (Table 2, Figure 1b; See covariances of exogenous predictors in Supplemental Material, Table S6), greater destructive IPC at Wave 1 was associated with higher maternal parenting entropy at Wave 2, even after controlling for Wave 1 maternal parenting entropy. Child ADHD symptoms at Wave 1 were associated with greater maternal parenting entropy at Wave 2. Once again, child ADHD symptoms exhibited moderate stability across Waves 1 and 3. Greater maternal parenting entropy at Wave 2 was linked to higher child ADHD symptoms at Wave 3, even after accounting for Wave 1 child symptoms and the role of other covariates (e.g., averaged maternal negative parenting). The indirect effect of greater destructive IPC leading to higher maternal entropy, and thereby higher child ADHD symptoms, achieved significance (Estimate = 0.05, 95% CI: [0.003, 0.11]).

#### Father models

In both the constructive and destructive IPC models (Table 3; See covariances of exogenous predictors in Supplemental Material, Table S6), we found significant stability of child ADHD symptoms between Waves 1 and 3. Additionally, greater averaged paternal negative parenting at Wave 2 was linked to higher paternal parenting entropy at Wave 2. Interparental conflict, whether constructive or destructive, was not associated with paternal parenting entropy, nor was paternal parenting entropy linked to child ADHD symptoms.

**Table 3.** Pathway findings for IPC, paternal parenting entropy, and child ADHD symptoms (*N* = 235)

	*B (SE)*	*Z*	*p*	*β*
***Constructive IPC model***				
**Child ADHD symptoms wave 3**				
Child ADHD symptoms wave 1	0.80 (0.06)	13.41	<.01	0.68
Paternal parenting entropy wave 2	0.01 (0.01)	0.64	.52	0.04
Constructive IPC wave 1	0.05 (0.07)	0.69	.49	0.04
Averaged paternal negative parenting wave 2	0.24 (0.16)	1.55	.12	0.09
Paternal parenting entropy wave 1	0.02 (0.01)	1.31	.19	0.07
**Paternal parenting entropy wave 2**				
Averaged paternal negative parenting wave 2	2.39 (1.19)	2.00	.05	0.20
Paternal parenting entropy wave 1	0.12 (0.08)	1.59	.11	0.12
Constructive IPC wave 1	−0.03 (0.39)	−0.08	.94	−0.01
Child ADHD symptoms wave 1	0.09 (0.41)	0.21	.83	0.02
***Destructive IPC model***				
**Child ADHD symptoms wave 3**				
Child ADHD symptoms wave 1	0.80 (0.06)	13.40	<.01	0.67
Paternal parenting entropy wave 2	0.01 (0.01)	0.67	.51	0.04
Destructive IPC wave 1	0.04 (0.08)	0.49	.63	0.02
Averaged paternal negative parenting wave 2	0.20 (0.16)	1.29	.20	0.08
Paternal parenting entropy wave 1	0.02 (0.01)	1.31	.19	0.07
**Paternal parenting entropy wave 2**				
Averaged paternal negative parenting wave 2	2.44 (1.16)	2.10	.04	0.20
Paternal parenting entropy wave 1	0.12 (0.08)	1.58	.11	0.12
Destructive IPC wave 1	−0.19 (0.64)	−0.29	.77	−0.02
Child ADHD symptoms wave 1	0.09 (0.41)	0.23	.82	0.02

### Constructive vs. Destructive IPC

We ran models contrasting the role of constructive vs. destructive IPC in relation to parenting entropy and child ADHD symptoms. As shown in Table 4 (See covariances of exogenous predictors in Supplemental Material, Table S6), in the mother’s model, *only constructive* IPC at Wave 1 was associated with lower maternal parenting entropy at Wave 2, after controlling for maternal parenting entropy at Wave 1. That said, after including constructive IPC in the model, the association between destructive IPC at Wave 1 and maternal parenting entropy was no longer significant. In addition, child ADHD symptoms at Wave 1 were linked to greater maternal parenting entropy at Wave 2. Furthermore, child ADHD symptoms showed moderate stability between Waves 1 and 3. Maternal parenting entropy at Wave 2 was again linked to higher ADHD symptoms at Wave 3, after controlling for symptoms at Wave 1. The indirect effect of greater constructive IPC in association with lower maternal parenting entropy, and thereby, lower child ADHD symptoms, remained significant (Estimate = −0.04, 95% CI: [−0.09, −0.0003]). In the father’s model, neither constructive nor destructive IPC was associated with paternal parenting entropy, and paternal parenting entropy was not linked to child ADHD symptoms.

**Table 4.** Pathway findings for constructive & destructive IPC, maternal and paternal parenting entropy, and child ADHD symptoms (*N* = *235)*

	*B (SE)*	*Z*	*p*	*β*
***Mother model: constructive & destructive IPC***				
**Child ADHD symptoms wave 3**				
Child ADHD symptoms wave 1	0.76 (0.06)	12.04	<.01	0.64
Maternal parenting entropy wave 2	0.03 (0.01)	2.34	.02	0.16
Destructive IPC wave 1	0.11 (0.11)	0.95	.34	0.06
Constructive IPC wave 1	0.09 (0.09)	1.03	.31	0.08
Averaged maternal negative parenting wave 2	−0.20 (0.15)	−1.27	.20	−0.08
Maternal parenting entropy wave 1	0.01 (0.01)	1.56	.12	0.07
**Maternal parenting entropy wave 2**				
Averaged maternal negative parenting wave 2	1.31 (1.24)	1.06	.29	0.11
Maternal parenting entropy wave 1	0.12 (0.07)	1.66	.10	0.12
Destructive IPC wave 1	0.66 (0.54)	1.23	.22	0.08
Constructive IPC wave 1	−1.06 (0.48)	−2.18	.03	−0.18
Child ADHD symptoms wave 1	0.72 (0.37)	1.96	.05	0.13
***Father model: constructive & destructive IPC***				
**Child ADHD symptoms wave 3**				
Child ADHD symptoms wave 1	0.80 (0.06)	13.44	<.01	0.68
Paternal parenting entropy wave 2	0.01 (0.01)	0.69	.49	0.04
Destructive IPC wave 1	0.11 (0.10)	1.04	.30	0.06
Constructive IPC wave 1	0.09 (0.09)	1.05	.30	0.08
Averaged paternal negative parenting wave 2	0.24 (0.16)	1.53	.13	0.09
Paternal parenting entropy wave 1	0.01 (0.01)	1.25	.21	0.06
**Paternal parenting entropy wave 2**				
Averaged paternal negative parenting wave 2	2.37 (1.20)	1.98	.05	0.20
Paternal parenting entropy wave 1	0.13 (0.08)	1.60	.11	0.12
Destructive IPC wave 1	−0.30 (0.80)	−0.38	.71	−0.04
Constructive IPC wave 1	−0.15 (0.50)	−0.30	.76	−0.03
Child ADHD symptoms wave 1	0.08 (0.41)	0.19	.85	0.01

### Sensitivity analyses

To examine the robustness of the findings we report about mothers, we performed several follow-up sensitivity tests. First, we included additional key demographic covariates (i.e., child age, gender, and family income-to-needs ratio measured at Wave 1) in the models examining the indirect effects of interparental conflict on child ADHD symptoms. As shown in the Supplemental Material (Table S3; see Table S5 for the correlation matrix among income-to-needs, interparental conflict, and parenting entropy), the findings remained unchanged. Both indirect pathways were significant in the originally reported directions: greater Wave 1 constructive interparental conflict was linked to lower maternal parenting entropy at Wave 2, and thereby, fewer child ADHD symptoms at Wave 3; conversely, greater Wave 1 destructive interparental conflict was linked to elevated maternal parenting entropy at Wave 2, and thereby, higher child ADHD symptoms at Wave 3.

In the second sensitivity test, appreciating the potential heritability of traits underlying ADHD symptoms, we included the maternal executive functioning measure as a covariate. This composite measure was derived from a battery of behavioral tasks assessing inhibitory control (Stop Signal Task; Verbruggen et al., 2008), set-shifting (Trail Making Task; Bowie & Harvey, 2006), and working memory (WAIS-IV Auditory Digit Span; Wechsler, 2008; see more details in Russotti et al., 2024). As detailed in Supplemental Table S4, our original findings regarding both constructive and destructive interparental conflict in association with maternal parenting entropy and child ADHD symptoms remained significant even when controlling for maternal executive functioning.

## Discussion

Utilizing a multi-method, multi-informant, and three-wave longitudinal design, we sought to evaluate how maternal and paternal parenting entropy may operate as indirect factors linking interparental conflict and child ADHD symptoms. Findings documented that constructive interparental conflict was associated with lower maternal parenting entropy, which in turn was associated with fewer child ADHD symptoms over time. Conversely, destructive interparental conflict was associated with higher maternal parenting entropy, which in turn was linked to more child ADHD symptoms over time. A follow-up test considering the role of both constructive and destructive conflict together indicated that only constructive conflict was significantly linked to lower maternal parenting entropy, and thereby, fewer child ADHD symptoms. In contrast to mothers, paternal parenting entropy was not related to interparental conflict, nor was the former linked to child ADHD symptoms. The present study advanced the literature in several ways. First, findings expanded our understanding of the antecedents of unpredictable parenting behavior, which itself functioned as critical indirect factors between broader family adversity (i.e., interparental conflict) and child psychopathology. Second, we demonstrated the soundness and feasibility of using the entropy approach to quantify the (unpredictable) pattern of parenting behavior across several aspects of molar-rated parenting behavior. Third, this study refines our understanding of parental unpredictability by examining the role of both maternal and paternal parenting entropy. Findings revealed a mother-specific effect, highlighting that maternal, but not paternal, parenting entropy is a key indirect factor linking interparental conflict and child ADHD symptoms.

Before delving into the findings, it is crucial to discuss the construct of parenting entropy and its validity. As we highlighted earlier, our approach to quantifying parenting entropy was inspired by a well-established procedure for measuring maternal mood entropy (e.g., Glynn et al., 2018; Howland et al., 2021). That said, although we did not utilize an established parenting survey, our approach of using multiple observational codings had distinct advantages. These codes, all reflecting the same underlying dimension of parenting and demonstrating excellent internal consistency, offered benefits such as reduced reporting bias and a more objective assessment of parenting behaviors. Tests of convergent validity provided support for the maternal parenting entropy, such that it was correlated, even though at a low to moderate range, with indices of greater parenting unpredictability, and lower positive aspects of parenting behavior, and higher negative aspects of parenting behavior. In addition, although evidence for paternal parenting entropy was slightly weaker, greater paternal parenting entropy was still associated with lower levels of positive parenting behavior and higher levels of negative parenting behavior. More specifically, paternal entropy was correlated in expected directions with variables such as technical scaffolding (correlated negatively), and authoritarian discipline (correlated positively). We proceeded with the analysis of paternal data for several reasons. First, the paternal entropy measure showed convergent validity; it demonstrated meaningful associations with observed parenting (see more details in Supplemental Material, Table S2). Second, as we elaborate below, the relatively low correlations among different indicators of unpredictability (e.g., entropy, residualized parenting variability, and dyadic parent–child unpredictability) likely reflect the multifaceted nature of the unpredictability construct, where different methodologies capture different aspects of environmental unpredictability rather than a lack of validity in a specific measure (Hollenstein et al., 2013; Walasek et al., 2024). Third, we believe that reporting these null findings for fathers is a necessary step to avoid publication bias in the relatively nascent field of paternal unpredictability. These results provide a foundation for future hypotheses regarding the unique role of fathers, though we advise readers to interpret the paternal findings within the context of this preliminary validity evidence.

Furthermore, the relatively low correlations among different indicators of parenting unpredictability (i.e., parenting entropy, residualized parenting variability, and dyadic parent–child unpredictability) for both parents align with prior findings showing nonsignificant or, at best, moderate correlations among various indices of environmental unpredictability (e.g., Walasek et al., 2024). Thus, although these indicators of parenting unpredictability are not highly correlated, parenting unpredictability is a complex construct that each indicator may capture a different aspect of it (e.g., unpredictability on a moment-to-moment basis, unpredictability across different aspects of parenting behavior, unpredictability across different interaction contexts; e.g., Hollenstein et al., 2013; Platts et al., 2025). Therefore, we encourage future work to consider different indicators of parenting unpredictability simultaneously and evaluate how these indicators may have different implications for the families and child development.

Turning to the study results, first, our findings that maternal parenting entropy operated as an indirect factor between both forms of interparental conflict and child ADHD symptoms align with theory and prior literature. Crucially, all findings emerged after controlling for the average level of maternal parenting behavior. This suggests that maternal parenting entropy (i.e., the unpredictable pattern of parenting) operates above and beyond the effects of the overall quality or level of parenting. In particular, our findings that destructive interparental conflict was linked to greater development in maternal parenting entropy, and that constructive interparental conflict was associated with lower growth in maternal parenting entropy over time was consitent with the spillover hypotheses and prior literature in documenting a negative impact of destructive (e.g., Krishnakumar & Buehler, 2000), and a protective role of constructive interparental conflict on parenting (Kopystynska et al., 2020; McCoy et al., 2013; Warmuth et al., 2020). Thus, constant exposure to anger and hostility within the interparental relationship may overwhelm the mothers, eroding their cognitive abilities (e.g., impaired executive functioning; Deater-Deckard & Sturge-Apple, 2017; Sturge-Apple et al., 2017), and evoking dysregulated physiological activities (e.g., Sturge-Apple et al., 2009) that may ultimately undermine mothers’ ability to parent effectively and consistently during the parent–child interactions. In contrast, supportive, perspective-taking, and problem-solving behaviors, even when parents are resolving a disagreement, not only do not impair cognitive functioning and physiological regulation but may also facilitate the cohesion and strength of the marital bond and coparenting alliances, and model strategies for problem-solving and perspective taking (e.g., Swerbenski et al., 2023), which are critical processes involved in parents’ consistent and effective parenting.

In turn, the finding that greater maternal parenting entropy was associated with greater development of child ADHD symptoms aligns with prior literature (e.g., Davis et al., 2019; Davis & Glynn, 2024; Holmberg et al., 2022; Li & Lansford, 2018). Thus, parents may provide crucial scaffolding as children develop the ability to control their behavior, impulses, and attention. Parents who are themselves depleted of mental and psychological resources may be unable to consistently and effectively regulate and discipline their children, particularly in the challenging forbidden-toy context (i.e., when children may be motivated to misbehave while parents are occupied with their own task). This inconsistency may impair the development of children’s effortful control, which can eventually manifest as elevated ADHD symptoms. Theoretically, these findings extend family systems frameworks by identifying parenting entropy as a key target of the spillover effect that eventually shape child development. While existing models posit that interparental conflict undermines parenting quality, our results suggest that this creates not just a deficit in warmth or sensitivity, but a dysregulation in the predictability of the parent–child signal. By conceptualizing entropy as a distinct pathway, our study supports the view that negativity or supportiveness within the interparental system may permeate the parent–child subsystem, manifesting as parents’ predictability during parent–child interactions. Importantly, sensitivity analyses indicated that our findings held across multiple sensitivity tests that accounted for various demographic factors, levels of parenting behavior, and parental executive functioning. These analyses confirm that the maternal parenting entropy measure provides unique predictive utility and functions as a robust factor above and beyond the confounding effects of multiple family, parent, and child factors (e.g., child age, gender, family socioeconomic resources, maternal executive functioning).

Second, despite the role of maternal parenting entropy, paternal parenting entropy was unrelated to either interparental conflict or child psychopathology. The finding that the development of paternal parenting entropy was not associated with interparental conflict is generally consistent with prior literature documenting that mothers are more vulnerable to the impact of interparental conflict (e.g., Sturge-Apple et al., 2009). That is, fathers might be less invested in close interparental relationships than mothers and place less emphasis on their role as spouses and parents, which, collectively, makes paternal parenting less shaped by interparental conflict (Thompson & Walker, 1989). Turning to child functioning, our findings also suggested a nonsignificant association between paternal parenting entropy with child ADHD symptoms. This might be accounted for by several reasons. To begin with, it is important to consider the null findings in the context of the weaker convergent validity of the paternal parenting entropy measures. As noted previously, while the paternal measure demonstrated meaningful associations with specific parenting behaviors (e.g., correlated negatively with technical scaffolding and positively with authoritarian discipline), its overall convergent validity was weaker than that of the maternal measure. This relatively lower level of validity may suggest that paternal parenting entropy, as quantified in this study, may not fully capture the specific nuances of unpredictable or disorganized paternal interactions that would lead to child ADHD symptoms. Other potential reasons may involve that paternal parenting entropy may be a more salient factor shaping other domains of child functioning in contrast to ADHD symptoms (e.g., risk-taking, social competence; Cabrera et al., 2018), or that the role of paternal parenting entropy may only emerge for children with certain temperamental traits (e.g., Li et al., 2023). Although these are plausible explanations, they are beyond the scope of the current study. Therefore, we encourage future research to further explore the differences and implications of maternal versus paternal parenting unpredictability.

Beyond these individual effects for each parent, an important consideration for future research is the potential for concordance or interaction between maternal and paternal unpredictability. For instance, it remains an open and compelling question whether a parent who is highly predictable in their behaviors can buffer the negative developmental impacts of a partner who is less predictable. While testing these interactive or “buffering” effects was beyond the scope of the current study, particularly as our design utilized separate dyadic tasks rather than a triadic setting where parenting behaviors could co-occur, we believe this is a critical next step for the field. Future work using triadic interaction tasks would be better positioned to evaluate how parents’ unpredictability might amplify or mitigate each other’s impact on children. Furthermore, exploring how the parental dyad functions as a unit (e.g., unpredictability at the “team” level) would provide a more systemic understanding of how family-level unpredictability may shape child psychopathology.

Third, when considering the role of constructive and destructive interparental conflict simultaneously with regard to maternal parenting entropy and child ADHD symptoms, only the effects of constructive, but not destructive conflict, were retained. Thus, even though the two forms of interparental conflict were moderately (and negatively) correlated, it seems that constructive conflict may help offset the risks of destructive conflict on parenting entropy. These findings align with prior work showing that constructive interparental conflict has a somewhat stronger association with inconsistent parenting than destructive interparental conflict does (e.g., Warmuth et al., 2020). Thus, even though the reformulated emotional security theory (EST-R) posits that threat cues within the interparental conflict (e.g., anger, hostility) may be more influential for child adjustment (e.g., Davies & Sturge-Apple, 2007), our findings suggest that the supportive and constructive aspects of the interparental conflict may be a more salient predictor for the *parents* (i.e., mothers) to function consistently and effectively within the parent–child subsystem.

Several limitations should be acknowledged despite the strengths of the present study. First, the sample primarily consisted of low- to middle-SES, two-parent families, which may limit the generalization of findings to high-risk populations. For instance, although parents in our sample demonstrated individual differences in destructive interparental conflict, these non-at-risk families still showed more variance in the constructive than in the destructive aspects of interparental conflict. This could explain the more salient effect of constructive interparental conflict on maternal parenting entropy when both forms of conflict behavior are considered together. Second, although we measured child ADHD symptoms using both parents’ reports, the assessment relied solely on a survey and included only several items (i.e., the Strengths and Difficulties Questionnaire). A more comprehensive assessment of ADHD symptoms via multiple methods (e.g., diagnostic interview) is encouraged in future research. Third, although we consider assessing parenting entropy during parent–child interaction a strength, our observation of parenting behavior was limited to two five-minute parent–child interactions in the laboratory and may not fully capture real-life parent–child interactions at home. Relatedly, although our interparental conflict paradigm is well established and broadly used in prior research (e.g., Sturge-Apple et al., 2009), it is possible that parents’ conflict behavior within the laboratory may not fully reflect how they resolve conflict in real-life settings. Furthermore, regarding potential spillover effects from the interparental conflict discussion into parent–child interactions, the order of parent–child tasks was counterbalanced across families. Specifically, a randomly selected half of the families completed the mother–child interaction first, while the other half completed the father–child interaction first. This randomization was used to ensure that there was no systematic bias in which one parent consistently followed the conflict task, thereby mitigating the concern that immediate spillover effects would artificially inflate or suppress effects for a specific parent. While the current study utilized counterbalancing to control for these effects, future research with larger samples could explicitly examine task order as a moderating factor to further clarify the duration and nature of spillover from interparental conflict into parent–child interactions.

Notwithstanding these limitations, the present study documented that maternal parenting entropy is an indirect factor that links interparental conflict and the development of child ADHD symptoms during early childhood. Findings broaden our understanding of the antecedents of unpredictable parenting behavior, which, in turn, has crucial implications for child development. Clinically, identifying parenting entropy as a mediator offers a novel and modifiable target for intervention. Traditional interventions often focus on reducing interparental conflict, which can be resistant to change. However, our findings suggest that enhancing parenting predictability may serve as a buffer even when conflict persists. Interventions could specifically target parenting “predictability” or “consistency” as therapeutic goals, coaching parents to maintain predictable disciplinary responses to reduce chaos in parent–child interactions. By helping parents stabilize their behavioral signals, clinicians may be able to interrupt the cascade from marital adversity to child maladjustment, effectively breaking the risk cascade between family adversity and child psychopathology.

## Supporting information

10.1017/S0954579426101655.sm001 Li et al. supplementary material Li et al. supplementary material

## Data Availability

Availability of Data and Methods/Materials: The data and materials necessary to reproduce the analyses presented here are not publicly accessible due to ethical considerations for protecting participant privacy and because the large longitudinal study is still active. Yet, the data can be requested from the corresponding author upon reasonable request. Availability of code: The analytic code presented in this paper is available in OSF (https://osf.io/4u8pd/overview).

## Supplementary material

The supplementary material for this article can be found at https://doi.org/10.1017/S0954579426101655.

## Preregistration statement

The study is exploratory and thus not preregistered.

## Table 2. Long description

The table presents the results of longitudinal autoregressive models examining the associations between constructive and destructive interparental conflict, maternal parenting, and child ADHD symptoms. It includes two main models: Constructive IPC model and Destructive IPC model. Each model has two rows of outcomes: Child ADHD symptoms wave 3 and Maternal parenting entropy wave 2. The table has 7 columns: B (SE), Z, p, and beta (β). The Constructive IPC model shows significant associations for Child ADHD symptoms wave 1, Maternal parenting entropy wave 2, and Constructive IPC wave 1 with Child ADHD symptoms wave 3. The Destructive IPC model shows significant associations for Child ADHD symptoms wave 1, Maternal parenting entropy wave 2, and Destructive IPC wave 1 with Child ADHD symptoms wave 3. Significant p-values are bolded, indicating statistical significance at conventional levels (p < .05).

Navigate back to Table 2..

## Table 1. Long description

The table presents descriptive information and bivariate correlations for primary study variables across two waves. It includes 10 rows and 10 columns, with row labels and column headers indicating different variables and their correlations. The table captures values such as correlations, means, standard deviations, minimum and maximum values, skewness, and kurtosis. Row 1: Constructive IPC wave 1. Row 2: Destructive IPC wave 1, -0.57**. Row 3: Maternal parenting entropy wave 1, -0.09, -0.04. Row 4: Maternal parenting entropy wave 2, -0.30**, 0.20**, 0.17*. Row 5: Paternal parenting entropy wave 1, 0.08, -0.001, -0.01, 0.12†. Row 6: Paternal parenting entropy wave 2, -0.05, -0.002, 0.02, -0.05, 0.13†. Row 7: Averaged maternal negative parenting wave 2, -0.43**, 0.17*, 0.12†, 0.22**, -0.15*, -0.02. Row 8: Averaged paternal negative parenting wave 2, -0.28**, 0.12†, 0.05, 0.18**, 0.05, 0.20**, 0.28**. Row 9: Child ADHD symptoms wave 1, -0.10, 0.04, 0.12†, 0.17*, 0.03, 0.01, 0.09, -0.06. Row 10: Child ADHD symptoms wave 3, -0.03, 0.05, 0.15*, 0.28**, 0.14*, 0.06, -0.02, 0.05, 0.68**. The table also includes statistical values such as N, Mean (SD), Min, Max, Skewness, and Kurtosis for each variable.

Navigate back to Table 1..
