# Supplementary material for: Constructive and destructive interparental conflict, parenting entropy, and child ADHD symptoms
Source: Dev Psychopathol. 2026 Jul 13:1–14. Online ahead of print. doi: 10.1017/S0954579426101655 (PMC13423604; doi:10.1017/S0954579426101655)
Supplement: Li et al. supplementary material [file S0954579426101655sup001.docx]

Supplemental Material

Table S1. Intraclass Correlation Coefficients for Maternal and Paternal Parenting Behavior at Waves 1 and 2.

|  | ICC  Maternal parenting  @ Wave 1 | ICC  Maternal parenting  @ Wave 2 | ICC  Paternal parenting  @ Wave 1 | ICC  Paternal parenting  @ Wave 2 |
| --- | --- | --- | --- | --- |
| Neglecting/Distancing | 0.74 | 0.88 | 0.87 | 0.92 |
| Harsh Discipline | 0.71 | 0.98 | 0.88 | 0.86 |
| Authoritarian Discipline | 0.69 | 0.95 | 0.83 | 0.97 |
| Technical Scaffolding (R)^a^ | 0.68 | 0.92 | 0.91 | 0.62 |
| Sensitivity (R) | 0.77 | 0.83 | 0.92 | 0.97 |
| Reasoning/Reminding (R) | 0.61 | 0.93 | 0.90 | 0.91 |
| Distracting (R) | 0.80 | 0.93 | 0.95 | 0.95 |

*Note.* a: The observational codes with **“(R)”** reflected that they were reverse scored (such that higher scores reflect more negative parenting) before creating the parenting entropy indicators.

Table S2. Correlation Matrix of Parenting Entropy and Parenting Behaviors for Each Parent at Each Wave. .

| Wave 1 Maternal Entropy & Parenting Behavior Correlation | | Wave 2 Maternal Entropy & Parenting Behavior Correlation | | Wave 1 Paternal Entropy & Parenting Behavior Correlation | | Wave 2 Paternal Entropy & Parenting Behavior Correlation | |
| --- | --- | --- | --- | --- | --- | --- | --- |
| **Parenting Variables** | Parenting Entropy Variable | **Parenting Variables** | Parenting Entropy Variable | **Parenting Variables** | Parenting Entropy Variable | **Parenting Variables** | Parenting Entropy Variable |
|  | **Maternal Parenting Entropy Wave 1** |  | **Maternal Parenting Entropy Wave 2** |  | **Paternal Parenting Entropy Wave 1** |  | **Paternal Parenting Entropy Wave 2** |
| 1. Maternal Technical Scaffolding Wave 1 | -.24** | 1. Maternal Technical Scaffolding Wave 2 | -.17* | 1. Paternal Technical Scaffolding Wave 1 | -.18** | 1. Paternal Technical Scaffolding Wave 2 | -.21** |
| 2. Maternal Sensitivity Wave 1 | -.35** | 2. Maternal Sensitivity Wave 2 | -.34** | 2. Paternal Sensitivity Wave 1 | -.06 | 2. Paternal Sensitivity Wave 2 | -.21** |
| 3. Maternal Reasoning/Reminding Wave 1 | -.18** | 3. Maternal Reasoning/Reminding Wave 2 | .25** | 3. Paternal Reasoning/Reminding Wave 1 | .15* | 3. Paternal Reasoning/Reminding Wave 2 | .13* |
| 4. Maternal Distracting Wave 1 | -.45** | 4. Maternal Distracting Wave 2 | -.33** | 4. Paternal Distracting Wave 1 | -.12† | 4. Paternal Distracting Wave 2 | -.30** |
| 5. Maternal Neglect/Distancing Wave 1 | .18** | 5. Maternal Neglect/Distancing Wave 2 | -.06 | 5. Paternal Neglect/Distancing Wave 1 | -.10 | 5. Paternal Neglect/Distancing Wave 2 | -.04 |
| 6. Maternal Harsh Discipline Wave 1 | -.08 | 6. Maternal Harsh Discipline Wave 2 | .11 | 6. Paternal Harsh Discipline Wave 1 | .02 | 6. Paternal Harsh Discipline Wave 2 | .06 |
| 7. Maternal Authoritarian Discipline Wave 1 | .30** | 7. Maternal Authoritarian Discipline Wave 2 | .30** | 7. Paternal Authoritarian Discipline Wave 1 | .24** | 7. Paternal Authoritarian Discipline Wave 2 | .20** |

Note. †*p*<.1, **p*< .05, ***p* <.01.

Table S3. *Pathway Findings for IPC, Maternal Parenting Entropy, and Child ADHD symptoms Controlling for Child Age, Gender, and Family Income-to-Needs Ratio(N=235).*

|  | ***B(SE)*** | ***Z*** | ***p*** | ***β*** |
| --- | --- | --- | --- | --- |
| ***Constructive IPC Model*** | | | | |
| **Child ADHD Symptoms Wave 3** | | | | |
| Child ADHD Symptoms Wave 1 | 0.70 (0.06) | 11.04 | < .001 | 0.59 |
| Maternal Parenting Entropy Wave 2 | 0.03 (0.01) | 2.26 | .02 | 0.15 |
| Constructive IPC Wave 1 | 0.02 (0.07) | 0.24 | .81 | 0.01 |
| Averaged Maternal Negative Parenting Wave 2 | -0.27 (0.14) | -1.88 | .06 | -0.10 |
| Maternal Parenting Entropy Wave 1 | 0.02 (0.01) | 1.73 | .08 | 0.07 |
| Child Age | 0.24 (0.25) | 0.96 | .34 | 0.04 |
| Child Gender | 0.84 (0.24) | 3.50 | < .001 | 0.18 |
| Family Income-to-Needs Ratio | -0.06 (0.08) | -0.82 | .41 | -0.05 |
| **Maternal Parenting Entropy Wave 2** | | | | |
| Child ADHD Symptoms Wave 1 | 0.58 (0.40) | 1.46 | .14 | 0.10 |
| Constructive IPC Wave 1 | -1.14 (0.40) | -2.87 | .004 | -0.19 |
| Averaged Maternal Negative Parenting Wave 2 | 0.75 (1.26) | 0.60 | .55 | 0.06 |
| Maternal Parenting Entropy Wave 1 | 0.09 (0.07) | 1.37 | .17 | 0.09 |
| Child Age | -2.49 (2.17) | -1.15 | .25 | -0.09 |
| Child Gender | 1.44 (1.50) | 0.96 | .34 | 0.06 |
| Family Income-to-Needs Ratio | -0.91 (0.46) | -1.98 | .048 | -0.14 |
| ***Destructive IPC Model*** | | | | |
| **Child ADHD Symptoms Wave 3** | | | | |
| Child ADHD Symptoms Wave 1 | 0.70 (0.06) | 11.02 | < .001 | 0.60 |
| Maternal Parenting Entropy Wave 2 | 0.03 (0.02) | 2.12 | .03 | 0.15 |
| Destructive IPC Wave 1 | 0.06 (0.09) | 0.63 | .53 | 0.03 |
| Averaged Maternal Negative Parenting Wave 2 | -0.27 (0.14) | -2.01 | .05 | -0.11 |
| Maternal Parenting Entropy Wave 1 | 0.02 (0.01) | 1.74 | .08 | 0.07 |
| Child Age | 0.27 (0.25) | 1.06 | .29 | 0.04 |
| Child Gender | 0.86 (0.23) | 3.71 | < .001 | 0.19 |
| Family Income-to-Needs Ratio | -0.05 (0.07) | -0.70 | .48 | -0.04 |
| **Maternal Parenting Entropy Wave 2** | | | | |
| Averaged Maternal Negative Parenting Wave 2 | 1.14 (1.22) | 0.93 | .35 | 0.09 |
| Maternal Parenting Entropy Wave 1 | 0.11 (0.07) | 1.59 | .11 | 0.11 |
| Destructive IPC Wave 1 | 1.15 (0.45) | 2.58 | .01 | 0.14 |
| Child ADHD Symptoms Wave 1 | 0.60 (0.40) | 1.52 | .13 | 0.11 |
| Child Age | -2.05 (2.30) | -0.89 | .37 | -0.07 |
| Child Gender | 0.76 (1.47) | 0.51 | .61 | 0.03 |
| Family Income-to-Needs Ratio | -1.25 (0.45) | -2.75 | .006 | -0.188 |

*Note*. Indirect pathway linking Wave 1 constructive interparental conflict and greater maternal parenting entropy at Wave 2, and thereby, greater child ADHD symptoms at Wave 3 was still significant (Estimate= -0.04, 95%CI: [-0.08, -0.003]. In addition, the indirect pathway linking Wave 1 destructive interparental conflict and greater maternal parenting entropy at Wave 2, and thereby, greater child ADHD symptoms at Wave 3, was also still significant (Estimate = 0.04, 95% CI: [0.0001, 0.09]).

Table S4. *Pathway Findings for IPC, Maternal Parenting Entropy, and Child ADHD symptoms Controlling for Maternal Executive Functioning (N=235).*

|  | ***B(SE)*** | ***Z*** | ***p*** | ***β*** |
| --- | --- | --- | --- | --- |
| ***Constructive IPC Model*** | | | | |
| **Child ADHD Symptoms Wave 3** | | | | |
| Child ADHD Symptoms Wave 1 | **0.76(0.06)** | **11.91** | **< .01** | **0.64** |
| Maternal Parenting Entropy Wave 2 | **0.04(0.01)** | **2.43** | **.02** | **0.17** |
| Constructive IPC Wave 1 | 0.04(0.07) | 0.64 | .53 | 0.04 |
| Averaged Maternal Negative Parenting Wave 2 | -0.16(0.16) | -1.02 | .31 | -0.06 |
| Maternal Parenting Entropy Wave 1 | 0.01(0.01) | 1.43 | .15 | 0.06 |
| Maternal Executive Functioning Wave 1 | 0.03(0.06) | 0.57 | .57 | 0.03 |
| **Maternal Parenting Entropy Wave 2** | | | | |
| Child ADHD Symptoms Wave 1 | 0.71(0.37) | 1.92 | .05 | 0.13 |
| Constructive IPC Wave 1 | **-1.31(0.40)** | **-3.32** | **< .01** | **-0.22** |
| Averaged Maternal Negative Parenting Wave 2 | 1.07(1.31) | 0.82 | .41 | 0.09 |
| Maternal Parenting Entropy Wave 1 | 0.11(0.07) | 1.59 | .11 | 0.11 |
| Maternal Executive Functioning Wave 1 | -0.21(0.35) | -0.60 | .55 | -0.04 |
| ***Destructive IPC Model*** | | | | |
| **Child ADHD Symptoms Wave 3** | | | | |
| Child ADHD Symptoms Wave 1 | **0.76(0.06)** | **11.86** | **< .01** | **0.64** |
| Maternal Parenting Entropy Wave 2 | **0.03(0.02)** | **2.23** | **.03** | **0.16** |
| Destructive IPC Wave 1 | 0.03(0.08) | 0.40 | .69 | 0.02 |
| Averaged Maternal Negative Parenting Wave 2 | -0.19(0.15) | -1.25 | .21 | -0.07 |
| Maternal Parenting Entropy Wave 1 | 0.01(0.01) | 1.38 | .17 | 0.06 |
| Maternal Executive Functioning Wave 1 | 0.04(0.06) | 0.68 | .50 | 0.04 |
| **Maternal Parenting Entropy Wave 2** | | | | |
| Averaged Maternal Negative Parenting Wave 2 | 1.71(1.24) | 1.38 | .17 | 0.14 |
| Maternal Parenting Entropy Wave 1 | 0.14(0.07) | 1.89 | .06 | 0.14 |
| Destructive IPC Wave 1 | **1.42(0.45)** | **3.16** | **< .01** | **0.17** |
| Child ADHD Symptoms Wave 1 | **0.72(0.37)** | **1.96** | **.05** | **0.13** |
| Maternal Executive Functioning Wave 1 | -0.35(0.34) | -1.03 | .30 | -0.07 |

*Note*. Indirect pathway linking Wave 1 constructive interparental conflict and greater maternal parenting entropy at Wave 2, and thereby, greater child ADHD symptoms at Wave 3 was still significant (Estimate= -0.05, 95%CI: [-0.10, -0.01]. In addition, the indirect pathway linking Wave 1 destructive interparental conflict to greater maternal parenting entropy at Wave 2 and, thereby, to greater child ADHD symptoms at Wave 3 also remained significant (Estimate = 0.05, 95% CI: [0.004, 0.11]).

Table S5. Correlation Matrix Involving Family Income-to-needs Ratio, Interparental Conflict, and Parenting Entropy.

| Variable | 1 | 2 | 3 | 4 | 5 | 6 | 7 |
| --- | --- | --- | --- | --- | --- | --- | --- |
| 1. Destructive IPC Wave 1 | - |  |  |  |  |  |  |
| 2. Constructive IPC Wave 1 | -.57** | - |  |  |  |  |  |
| 3. Family Income-to-Needs Ratio | -.16* | .44** | - |  |  |  |  |
| 4. Maternal Parenting Entropy Wave 2 | .20** | -.30** | -.30** | - |  |  |  |
| 5. Maternal Parenting Entropy Wave 1 | -.04 | -.09 | -.19** | .17* | - |  |  |
| 6. Paternal Parenting Entropy Wave 2 | .00 | -.05 | -.05 | -.05 | .02 | - |  |
| 7. Paternal Parenting Entropy Wave 1 | .00 | .08 | .02 | .12† | -.01 | .13† | - |

Table S6. Covariance Table in Primary Analyses.

|  | *Estimate(SE)* | *Z* | *p* |
| --- | --- | --- | --- |
| ***Mother Model: Constructive IPC Model (First Model in Table 2)*** | | | |
| Child ADHD Symptoms Wave 1~~ |  |  |  |
| Constructive IPC Wave 1 | -0.33(0.23) | -1.43 | .15 |
| Averaged Maternal Negative Parenting Wave 2 | 0.16(0.12) | 1.36 | .17 |
| Maternal Parenting Entropy Wave 1 | 2.50(1.58) | 1.58 | .12 |
| Constructive IPC Wave 1~~ |  |  |  |
| Averaged Maternal Negative Parenting Wave 2 | -0.74(0.12) | -6.12 | .00 |
| Maternal Parenting Entropy Wave 1 | -1.95(1.47) | -1.33 | .18 |
| Averaged Maternal Negative Parenting Wave 2~~ |  |  |  |
| Maternal Parenting Entropy Wave 1 | 1.07(0.74) | 1.46 | .15 |
| ***Mother Model: Destructive IPC Model (Second Model in Table 2)*** | | | |
| Child ADHD Symptoms Wave 1~~ |  |  |  |
| Destructive IPC Wave 1 | 0.11(0.16) | 0.70 | .48 |
| Averaged Maternal Negative Parenting Wave 2 | 0.16(0.12) | 1.37 | .17 |
| Maternal Parenting Entropy Wave 1 | 2.52(1.58) | 1.59 | .11 |
| Destructive IPC Wave 1~~ |  |  |  |
| Averaged Maternal Negative Parenting Wave 2 | 0.20(0.09) | 2.33 | .02 |
| Maternal Parenting Entropy Wave 1 | -0.66(1.00) | -0.66 | .51 |
| Averaged Maternal Negative Parenting Wave 2~~ |  |  |  |
| Maternal Parenting Entropy Wave 1 | 1.20(0.74) | 1.64 | .10 |
| ***Father Model: Constructive IPC Model (First Model in Table 3)*** | | | |
| Child ADHD Symptoms Wave 1~~ |  |  |  |
| Constructive IPC Wave 1 | -0.36(0.23) | -1.53 | .13 |
| Averaged Paternal Negative Parenting Wave 2 | -0.10(0.11) | -0.92 | .36 |
| Paternal Parenting Entropy Wave 1 | 0.68(1.37) | 0.50 | .62 |
| Constructive IPC Wave 1~~ |  |  |  |
| Averaged Paternal Negative Parenting Wave 2 | -0.48(0.12) | -3.87 | .00 |
| Paternal Parenting Entropy Wave 1 | 1.64(1.35) | 1.21 | .23 |
| Averaged Paternal Negative Parenting Wave 2~~ |  |  |  |
| Paternal Parenting Entropy Wave 1 | 0.43(0.81) | 0.53 | .60 |
| ***Father Model: Destructive IPC Model(Second Model in Table 3)*** | | | |
| Child ADHD Symptoms Wave 1~~ |  |  |  |
| Destructive IPC Wave 1 | 0.11(0.16) | 0.74 | .46 |
| Averaged Paternal Negative Parenting Wave 2 | -0.10(0.11) | -0.91 | .37 |
| Paternal Parenting Entropy Wave 1 | 0.68(1.37) | 0.50 | .62 |
| Destructive IPC Wave 1~~ |  |  |  |
| Averaged Paternal Negative Parenting Wave 2 | 0.15(0.09) | 1.70 | .09 |
| Paternal Parenting Entropy Wave 1 | -0.01(0.91) | -0.01 | .99 |
| Averaged Paternal Negative Parenting Wave 2~~ |  |  |  |
| Paternal Parenting Entropy Wave 1 | 0.47(0.81) | 0.58 | .56 |
| ***Mother Model: Constructive & Destructive IPC* (First Model of Table 4, Covariance)** | | | |
| Child ADHD Symptoms Wave 1~~ |  |  |  |
| Destructive IPC Wave 1 | 0.11(0.16) | 0.70 | .48 |
| Averaged Maternal Negative Parenting Wave 2 | 0.16(0.12) | 1.36 | .17 |
| Maternal Parenting Entropy Wave 1 | 2.53(1.58) | 1.59 | .11 |
| Destructive IPC Wave 1~~ |  |  |  |
| Averaged Maternal Negative Parenting Wave 2 | 0.20(0.09) | 2.40 | .02 |
| Maternal Parenting Entropy Wave 1 | -0.67(1.00) | -0.67 | .51 |
| Averaged Maternal Negative Parenting Wave 2~~ |  |  |  |
| Maternal Parenting Entropy Wave 1 | 1.05(0.74) | 1.42 | .16 |
| Child ADHD Symptoms Wave 1~~ |  |  |  |
| Constructive IPC Wave 1 | -0.33(0.23) | -1.43 | .15 |
| Constructive IPC Wave 1~~ |  |  |  |
| Averaged Maternal Negative Parenting Wave 2 | -0.75(0.12) | -6.12 | .00 |
| Maternal Parenting Entropy Wave 1 | -1.91(1.47) | -1.30 | .19 |
| Destructive IPC Wave 1~~ |  |  |  |
| Constructive IPC Wave 1 | -1.44(0.21) | -6.90 | .00 |
|  |  |  |  |
| ***Father Model: Constructive & Destructive IPC* (Second Model of Table 4, Covariance)** | | | |
| Child ADHD Symptoms Wave 1~~ |  |  |  |
| Destructive IPC Wave 1 | 0.11(0.16) | 0.74 | .46 |
| Averaged Paternal Negative Parenting Wave 2 | -0.10(0.11) | -0.95 | .34 |
| Paternal Parenting Entropy Wave 1 | 0.67(1.37) | 0.49 | .62 |
| Destructive IPC Wave 1~~ |  |  |  |
| Averaged Paternal Negative Parenting Wave 2 | 0.15(0.09) | 1.75 | .08 |
| Paternal Parenting Entropy Wave 1 | -0.01(0.91) | -0.01 | .99 |
| Averaged Paternal Negative Parenting Wave 2~~ |  |  |  |
| Paternal Parenting Entropy Wave 1 | 0.42(0.80) | 0.52 | .61 |
| Child ADHD Symptoms Wave 1~~ |  |  |  |
| Constructive IPC Wave 1 | -0.36(0.23) | -1.53 | .13 |
| Constructive IPC Wave 1~~ |  |  |  |
| Averaged Paternal Negative Parenting Wave 2 | -0.48(0.12) | -3.86 | .00 |
| Paternal Parenting Entropy Wave 1 | 1.64(1.35) | 1.21 | .23 |
| Destructive IPC Wave 1~~ |  |  |  |
| Constructive IPC Wave 1 | -1.44(0.21) | -6.90 | .00 |


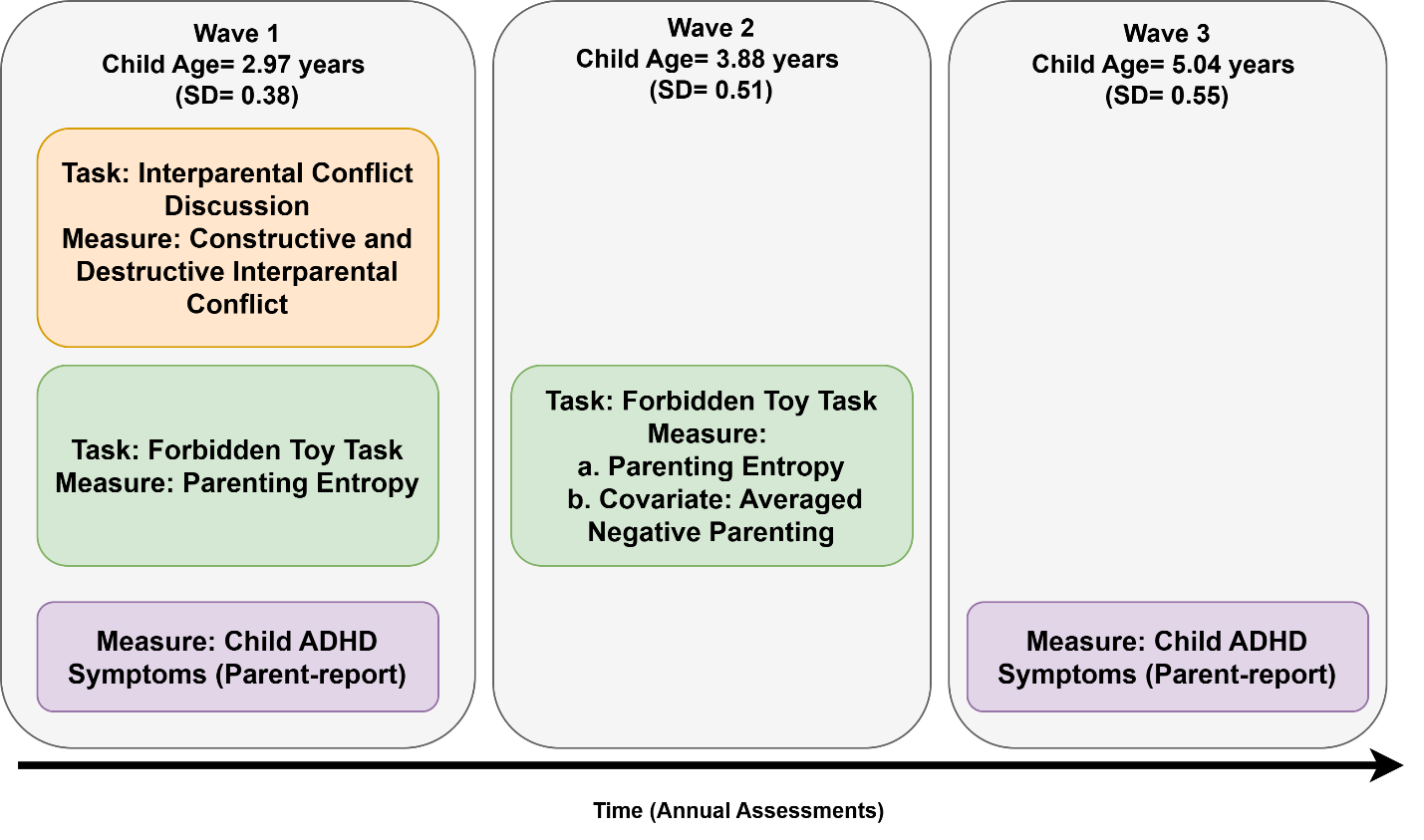


*Figure S1*. Overview of Study Design and Assessment Timeline.
